# COVID-19–Related Disruptions and Increased mHealth Emergency Use Intention: Experience Sampling Method Study

**DOI:** 10.2196/20642

**Published:** 2020-12-30

**Authors:** Zhenduo Zhang, Li Zhang, Junwei Zheng, Huan Xiao, Zhigang Li

**Affiliations:** 1 School of Management Harbin Institute of Technology Harbin China; 2 Department of Construction Management Kunming University of Science and Technology Kunming China; 3 School of Economics and Management Beijing Polytechnic Beijing China

**Keywords:** mobile health services, emergency use intention, event disruption, COVID-19–induced strain, promotion regulatory focus, mHealth, COVID-19

## Abstract

**Background:**

The COVID-19 pandemic has become a global public health event, which has raised concerns regarding individuals’ health. Individuals need to cope with the COVID-19 pandemic with guidelines on symptom recognition, home isolation, and maintain mental health. Besides routine use of mobile health (mHealth) such as accessing information to keep healthy, individuals can use mHealth services in situations requiring urgent medical care, which is defined as mHealth emergency use. It is not known whether individuals have increased their daily mHealth services emergency use as a result of disruptions caused by the COVID-19 pandemic.

**Objective:**

The purpose of this diary analysis study is to assess the influences of daily disruptions related to the COVID-19 pandemic on individuals’ mHealth emergency use. The secondary purpose of this study is to explore the mediating role of COVID-19–induced strain and the moderating role of promotion regulatory focus in the relationship between daily disruptions of COVID-19 and mHealth emergency use. Drawing from the cognitive activation theory of stress, we investigated the underlying mechanism and boundary condition of the influence of COVID-19–related disruptions on daily mHealth emergency use.

**Methods:**

To test the proposed model, this study adopts the experience sampling method to collect daily data. The experience sampling method helps researchers to capture participants’ fluctuations in emotions, mental engagement in an activity, and experienced stress. This study collected 550 cases nested in 110 samples in mainland China to test the conceptual model. In addition, we employed hierarchical linear modeling analysis to test the effect of COVID-19–related disruptions on mHealth emergency use.

**Results:**

We found that COVID-19–related disruptions increased COVID-19–induced strain (γ=0.24, *P*<.001) and mHealth emergency use on a daily basis (γ=0.28, *P*<.001). COVID-19–induced daily strain mediated this relationship (effect=0.09, 95% CI 0.05-0.14). Promotion regulatory focus moderated the relationship between COVID-19–induced strain and mHealth emergency use (γ=0.35, *P*=.02). In addition, the indirect relationship between disruptions and mHealth emergency use intentions through COVID-19–induced strain is contingent upon promotion regulatory focus: this relationship was stronger in those with high promotion regulatory focus (effect=0.12, 95% CI 0.06-0.19) than in those with low promotion regulatory focus (effect=0.06, 95% CI 0.02-0.11).

**Conclusions:**

Event disruption of the COVID-19 pandemic induced mHealth emergency use intention through increased psychological strain. Furthermore, individuals’ promotion regulatory focus amplified this indirect relationship. Our findings extend our understanding of the factors underlying mHealth emergency use intention and illustrate the potential contingent role of promotion regulatory focus in the cognitive activation theory of stress. This study also opens avenues for future research on mHealth emergency use intention in other countries and cultural settings.

## Introduction

### Background

The COVID-19 pandemic has become a worldwide public health event. This has resulted in greater concerns regarding one’s health and well-being [1]. Similar to the research of Morgeson et al [2], this study considers event disruption as a negative influence on behaviors of health information system use. Event disruption is defined as a discontinuity in the environment where the external situation has somehow changed [3]. The COVID-19 pandemic has likely transformed people’s routines and, thus, requires an additional investment of resources to adapt effectively to cope with increased health concerns [4].

Mobile health (mHealth) service is defined as health care practice supported by mobile devices. Given that our research focuses on the mHealth service in the COVID-19 pandemic, the mHealth service in this study includes apps that health care professionals use to treat clinical disease, reinforce treatment adherence, provide consultation to the users, and educate users on self-monitoring of the disease COVID-19 [5]. mHealth service is an essential component of health information technology, which has the potential to enhance health care quality and promote good health [6-8]. Besides routine use, individuals use mHealth services in situations requiring urgent medical care, which is defined as mHealth emergency use [8]. The aims of mHealth emergency use are to help users to acquire appropriate care in urgent situations and improve the efficiency of treatment toward specific disease [9]. mHealth services can facilitate the fast delivery of health care information to the users, assisting users to make medical decisions in emergencies [8]. The outbreak of the COVID-19 pandemic is a serious public health event and threatens everyone’s health. However, it is not yet known whether the COVID-19 pandemic influences mHealth emergency use. Furthermore, the pandemic situation changes on a daily basis, resulting in corresponding daily changes in disruptions and their effects. Therefore, our first research question asks whether event disruption of the COVID-19 pandemic increases daily mHealth emergency use*.*

The cognitive activation theory of stress (CATS) [10,11] addresses the role of cognitive appraisal and interpretation in shaping the way an individual responds to stressful events [12]. When the event is regarded as threatening and challenging, the individual may experience strain, which is defined as an unpleasant subjective experience toward a specific event [13]. Considering the disruptions caused by the COVID-19 pandemic, the strain will likely be induced and consequently impact an individual’s attitudes and preferred coping strategies [14]. Our second research question is whether psychological strain mediates the relationship between event disruption of the COVID-19 pandemic and mHealth emergency use on a daily basis.

Whether an individual decides to use mHealth to cope with COVID-19–induced strain is contingent upon their preferred method to deal with problems [15]. Prior research has demonstrated the critical role of promotion regulatory focus in facilitating individuals’ adoption of new technologies [16,17]. Promotion regulatory focus denotes a sensitivity to gains and a desire for advancement and growth [18]. Individuals with strong promotion regulatory focus have greater intentions to use mHealth to help cope with COVID-19–induced strain. The third research question of this study is to investigate whether the indirect relationship between event disruption and mHealth emergency use intention through COVID-19–induced strain is contingent upon individuals’ promotion regulatory focus*.*

**Figure 1:**
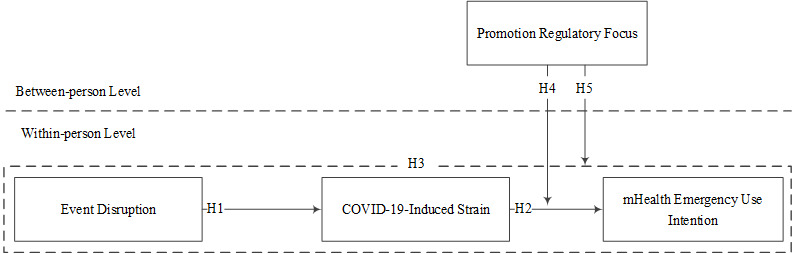
Conceptual model. H: hypothesis; mHealth: mobile health.

To address our three research questions, we used the experience sampling method to test the conceptual model (see Figure 1). Our study has three potential contributions to mHealth literature. First, this study examines the temporal relationship between the event disruption of the COVID-19 pandemic and mHealth emergency use intention. Second, this study explores the underlying mechanism through which the event disruption impacts mHealth emergency use intention by examining the mediating role of COVID-19–induced strain. Third, this study depicts the boundary condition under which event disruption is more or less influential on mHealth emergency use intention through COVID-19–induced strain by exploring the moderating role of promotion regulatory focus.

### Literature Review of CATS

CATS proposes that stress occurs with a discrepancy between desired outcomes and reality [19]. Individuals will feel stress when the future is unpredictable or their resources are not sufficient to cope with the demands [11], whereas if individuals have control over the situations to achieve favorable outcomes, the stress will not occur [20]. Cognitive appraisal is the central role of cognitive activation [11]. Primary and secondary appraisals are two sequential stages in the cognitive appraisal process [21]. In the primary appraisal stage, individuals will evaluate the target events as harmful or beneficial. If such events are regarded as harmful, individuals will then evaluate the extent that they can overcome such harmful events in the secondary appraisal [22]. When they cannot handle the stressful events, the strain would arise. Individuals are motivated to take the necessary strategies and change their attitudes to get rid of strain [23]. Especially when individuals anticipate that their chosen responses to stressful events will be associated with a favorable outcome, they are coping [11].

CATS offers us a framework to elaborate on the influences of the COVID-19 pandemic on individuals’ mHealth emergency use. The unpredictable and detrimental characteristics of the COVID-19 pandemic change individuals’ life and work. Confronted with such changes, the strain will arise in individuals and further shape their attitudes and coping behavior. mHealth is an effective instrument to realize disease prevention and health promotion [24], which helps individuals to successfully cope with the COVID-19 pandemic. Therefore, this study adopts CATS as the overarching theory to explain the indirect relationship between the COVID-19 pandemic and mHealth emergency use intention through COVID-19–induced strain.

### Hypothesis Development

#### Event Disruption, mHealth Emergency Use Intention, and COVID-19–Induced Strain

When events are urgent, unpredictable, unexpected, and threatening, they are regarded as stressful and may result in negative psychological, physical, and physiological outcomes [25]. CATS posits that strain or stress experience arises from challenging stressful events [26,27].

This corresponds with the event disruptions of the COVID-19 pandemic, which reflect change and discontinuity of the external situation [2]. The COVID-19 pandemic has significantly changed the way individuals live and work. For instance, Chinese citizens are required to quarantine and work from home [28]. The COVID-19 pandemic has transformed daily routines, requiring individuals to invest considerable resources and energies to adapt effectively. Risk of exposure to the virus, uncertainty about income, shortages of protective equipment, and irregular work hours have all contributed to the increase in stress experience or strain [29,30]. Additionally, the COVID-19 pandemic situation changes on a daily basis, and therefore, the effects on stress levels also change on a daily basis. Taken together, we hypothesize that:

- Hypothesis (H)1: Event disruption is positively associated with COVID-19–induced strain on a daily basis.

CATS links stressful events with coping behavior [11]. Stress coping refers to the constant adaptation of cognitive and behavioral efforts to deal with specific demands deriving from external or internal situations [31]. Two determinants of coping behavior are the specific context of the stressful situation and the individual’s expectations about the outcomes [11,31].

Regarding the context of stress, an individual’s coping response depends on their appraisal of the demands and resources available to handle the stressful event [32]. COVID-19–induced strain mainly arises from the uncertainty of infection and the risk of exposure to the virus [29]. To maintain their health status and prevent unexpected infection, individuals are driven to seek relevant information and help from professional medical personnel [33], which can be accommodated by mHealth. Through integrating advanced technology, mHealth can be accessed using portable and wireless communication equipment such as a tablet, mobile phone, or wearable device [8]. The remote and instant availability of mHealth provides convenience for recipients, especially during the COVID-19 pandemic [34].

In terms of expectations, the choice of coping behavior is determined by an individual’s anticipated outcome [8]. If desirable outcomes are expected, individuals are more likely to choose positive coping strategies in response to stressful experiences [35]. The COVID-19–induced strain has increased public health concerns. The uncertainty and unpredictability of COVID-19 require that individuals access instant and accurate information. Appropriate consultation and treatment can be provided via mHealth in situations of urgency [36]. Research has demonstrated the vital role that mHealth plays in the implementation of prehospital measures in response to specific diseases [37]. It effectively facilitates the delivery of health care services and accurate health-related information [38]. Based on these findings, we infer that mHealth is a preferred coping tool for daily COVID-19–induced strain. Therefore, we hypothesize the following:

- H2: COVID-19–induced strain is positively correlated with mHealth emergency use intention on a daily basis.

The event disruption of the COVID-19 pandemic makes the external situations unpredictable and uncertain. The changes in life induce stress experience in individuals. To cope with event disruption and COVID-19–induced strain on a daily basis, individuals are more likely to use mHealth in urgent situations to promote health status and prevent a specific disease. In this vein, we further hypothesize that:

- H3: COVID-19–induced strain mediates the relationship between event disruption and daily mHealth emergency use intention.

#### Moderating Role of Promotion Regulatory Focus

CATS suggests that the choice of coping behavior is determined by the interaction of personal and contextual variables [31]. Regulatory focus is regarded as one of the most important personality variables impacting coping behavior [15] and explains the motivations underlying goal setting [16]. The literature divides regulatory focus into two categories: promotion and prevention focus [39]. An individual with high prevention regulatory focus tries to ensure that they meet the minimum requirements, whereas those with high promotion regulatory focus strive to optimize the situation to achieve ideals and nurturance [40]. Research has shown that promotion regulatory focus plays a critical role in facilitating coping with stress positively [41]. Given that the use of mHealth is an active effort toward resolving COVID-19–induced strain, we adopted promotion rather than prevention regulatory focus to represent a personal preference of coping strategies.

In the context of COVID-19–induced strain, individuals with high promotion regulatory focus may regulate their actions and attitudes to achieve favorable outcomes [42] by generating potential approaches and strategies. When experiencing COVID-19–induced strain, they may seek immediate access to accurate medical information and health care services to reduce their health concerns as well as those of people who are close to them [29]. mHealth may be particularly suited to these individuals because of its timeliness and accessibility. In contrast, individuals with low promotion regulatory focus will not prioritize an optimal outcome and therefore will not consider access to health care services as urgent. These individuals are less likely to use mHealth to help cope with COVID-19–induced strain. Therefore, we hypothesize the following:

- H4: Promotion regulatory focus will moderate the relationship between daily COVID-19–induced strain and mHealth emergency use intention, such that the relationship is stronger in the condition of high promotion regulatory focus than in the condition of low promotion regulatory focus.

As previously mentioned, event disruptions of the COVID-19 pandemic cause unpredictable and unfavorable changes in personal and work life, which elevates stress experience. This induced strain may drive individuals to use mHealth in urgent situations, especially those with high promotion regulatory focus, as this will allow them to promote good health and prevent disease. We hypothesize that:

- H5: Promotion regulatory focus will moderate the indirect relationship between event disruption and daily mHealth emergency use intention through COVID-19–induced strain, such that the indirect relationship is stronger in the condition of high promotion regulatory focus than in the low promotion regulatory focus.

## Methods

### Data Collection

Based on the research of Du et al [43], we used convenience sampling to recruit our participants. We sought help from the administrative staff at the university. With their help, we contacted the alumni who updated their contact information within 2 years. We recruited the participants through the alumni networks of the public university in China. Through this convenience sampling method, we invited the participation of the alumni living or working in diverse areas in China, thereby bolstering the external validity of the research findings. The inclusion criteria included having a certain degree of smartphone use experience (≥1 year), living or working in mainland China, and having a mobile phone or tablet connected to the internet. The WeChat smartphone app was adopted for this study because of its popularity in China. A research group was created on WeChat, with an initial invitation to 150 potential participants to join the group.

The data collection contained two stages. On February 23, 2020, participants were asked to complete a baseline questionnaire regarding demographic information (gender, age, education) and promotion regulatory focus. From February 24 to 28, 2020, participants were sent a website link at 11 AM that assessed event disruptions and at 5 PM that assessed COVID-19–induced strain and mHealth emergency use intention on each day. Participants were asked to complete the questionnaires within 2 hours. Of the 150 individuals invited, we collected 550 matched responses from a total of 110 participants, yielding an effective response rate of 73.3%. The 110 participants received a ¥25 (about US $3.53) inconvenience allowance.

### Measurement Development

All of the measures of the constructs were developed based on previous research. We adapted each item to fit the daily gathering of data. For instance, one item of the original work strain scale is “I often feel too tense due to my work.” We adapted it as “Due to COVID-19 Pandemic, I lived and worked under a great deal of tension today” to fit the COVID-19 pandemic and the daily research context. Specifically, in accordance with suggestions from Donald et al [44], short scales were adopted to minimize the nonresponse rate. A 7-point Likert scale was used, ranging from 1 (*strongly disagree*) to 7 (*strongly agree*). The complete scale is shown in Multimedia Appendix 1.

### Daily Measurement

#### Event Disruptions

Measures for event disruptions were adapted from four items developed by Morgeson et al [2]. The sample item was “Today, COVID-19 pandemic disrupted my ability to get its work done.” The average score of this scale ranged from 1 to 7. The scale yielded a Cronbach alpha of .93.

#### COVID-19–Induced Strain

The COVID-19–induced strain was measured by three items adapted from the scale developed by House and Rizzo [45]. The sample item was “Due to COVID-19 Pandemic, I lived and worked under a great deal of tension today.” The range of the average score was from 1 to 7. The reliability of this scale was .76.

#### Emergency Use Intention

mHealth emergency use intention was measured by three items developed by Liu et al [8]. The sample item was “Today, I intended to use mHealth services under urgent medical requirements.” The range of the score was from 1 to 7. The Cronbach alpha of this scale was .92.

### Baseline Measurement

#### Promotion Regulatory Focus

The regulatory focus has been regarded as a personality trait, which is stable and not probable to change in a short time. Thus, this study put promotion regulatory focus at the baseline measurement [46-48]. Promotion regulatory focus was measured by nine items developed by Lockwood et al [49]. The sample item of this scale was “I frequently imagine how I will achieve my hopes and aspirations.” The average score of this scale ranged from 1 to 7. The alpha of this scale was .93.

#### Control Variables

We also collected demographic data including gender, education, age, and chronic disease, as they may influence mHealth use intention [8,50].

### Analytical Strategy

The data was nested, as the data were collected using the experience sampling method. The data had a two-level hierarchical structure, where daily level or within-person level data was positioned at level one and individual level or between-person level data was positioned at level two [51]. We, therefore, used hierarchical linear modeling (HLM) for our analyses [52].

The analysis contained two stages. First, we investigated the within-person level variance in the daily variables. The results showed about a 71%-85% variance for the within-person level for event disruption, COVID-19–induced strain, and mHealth emergency use intention, justifying the use of HLM. Second, we performed HLM (version 6.08) using a restricted maximum likelihood estimation for the parameter analyses. We conducted a moderated mediation model analysis with a random slope and used robust estimators in level one to indicate the daily or within-person effect. The daily variables (event disruption, COVID-19–induced strain, and mHealth emergency use intention) were group-centered.

## Results

### Participants

Table 1 shows the results of demographic information. Among the 110 participants, 54.5% (n=60) were males. Of the participants, 0.9% (n=1) only received primary school education, 1.8% (n=2) graduated from junior school, 27.3% (n=30) graduated from senior high school, 23.6% (n=26) held college certificates, and 46.4% (n=51) held Bachelor’s degrees or above. Regarding chronic disease, 88.4% (n=97) of the participants did not have a chronic disease. For the distribution of age, 1.8% (n=2) of the participants were younger than 26 years, 45.5% (n=50) ranged from 26 to 35 years, 32.7% (n=36) ranged from 36 to 45 years, and 20% (n=22) were older than 45 years.

**Table 1:** Participants’ demographic data (N=110).

Characteristic		Participants, n (%)
**Gender**		
	Male	60 (54.5)
	Female	50 (45.5)
**Chronic disease**		
	No	97 (88.4)
	Yes	13 (11.6)
**Education**		
	Primary school	1 (0.9)
	Senior school	2 (1.8)
	High school	30 (27.3)
	College	26 (23.6)
	Bachelor’s and above	51 (46.4)
**Age (years)**		
	<26	2 (1.8)
	26-35	50 (45.5)
	36-45	36 (32.7)
	≥46	22 (20.0)

### Multilevel Confirmatory Analysis

Given that our daily data were nested, we adopted multilevel confirmatory factor analysis rather than exploratory factor analysis to test the validity of the measurements and the common method variance [43,53]. The results showed that the proposed four-factor model yielded a better model fit (*χ*^2^_57_=151.22; root mean square error of approximation 0.06; comparative fit index 0.96; Tucker–Lewis index 0.95; standardized root mean square residual 0.03) than any other model. The results are shown in Table 2.

**Table 2:** Results of multilevel confirmatory factor analysis.

Models	Chi-square (*df*)	△ chi-square	*P* value	RMSEA^a^	CFI^b^	TLI^c^	SRMR^d^ (within)
EU^e^, ED^f^, LS^g^, PF^h^	151.22 (57)	N/A^i^	N/A	0.06	0.96	0.95	0.03
EU+ED, LS, PF	451.15 (59)	299.93	<.001	0.11	0.84	0.78	0.19
EU+LS, ED, PF	420.60 (59)	269.38	<.001	0.11	0.85	0.80	0.12
EU, LS+ED, PF	440.35 (59)	289.13	<.001	0.11	0.85	0.79	0.16
EU+LS+ED, PF	658.05 (60)	506.83	<.001	0.14	0.76	0.67	0.21

^a^RMSEA: root mean square error of approximation.

^b^CFI: comparative fit index.

^c^TLI: Tucker–Lewis index.

^d^SRMR: standardized root mean square residual.

^e^EU: mobile health emergency use intention.

^f^ED: event disruption.

^g^LS: COVID-19–induced strain.

^h^PF: promotion regulatory focus.

^i^N/A: not applicable.

### Descriptive Statistics

Tables 3 and 4 show the means, SDs, reliabilities, and correlations of the variables both at the within- and between-person levels. The results showed that event disruption was significantly correlated with daily mHealth emergency use (*r*=0.20, *P*<.001) and daily COVID-19–induced strain (*r*=0.29, *P*<.001). Event disruption was associated with daily COVID-19–induced strain (*r*=0.27, *P*<.001).

**Table 3:** Within-person level (N=550) means, SDs, and correlations.

Variables		Mean (SD)	1	2	3
**1. mHealth^a^ emergency use intention^b^**		4.84 (0.99)			
	*r*		1		
	*P* value		N/A^c^		
**2. Event disruption^d^**		3.81 (0.69)			
	*r*		0.20	1	
	*P* value		<.001	N/A	
**3. COVID-19–induced strain^e^**		4.20 (0.77)			
	*r*		0.29	0.27	1
	*P* value		<.001	<.001	N/A

^a^mHealth: mobile health.

^b^Cronbach alpha=.92.

^c^N/A: Not applicable.

^d^Cronbach alpha=.93.

^e^Cronbach alpha=.76.

**Table 4:** Between-person level (N=110) means, SDs, and correlations.

Variables			Mean (SD)		1		2		3		4		5
**1. Gender**			1.45 (0.50)										
	*r*			1									
	*P* value			N/A^a^									
**2. Education**			4.13 (0.94)										
	*r*			0.68		1							
	*P* value			<.001		N/A							
**3. Chronic disease**			1.12 (0.32)										
	*r*			0.06		0.07		1					
	*P* value			.19		.12		N/A					
**4. Age**			2.71 (0.80)										
	*r*			–0.49		–0.24		–0.16		1			
	*P* value			<.001		<.001		<.001		N/A			
**5. Promotion regulatory focus^b^**			4.08 (0.35)										
	*r*			0.03		–0.15		–0.02		–0.15		1	
	*P* value			.69		<.001		.57		<.001		N/A	

^a^N/A: Not applicable.

^b^Cronbach alpha=.93.

### Hierarchical Linear Modeling

Daily event disruption had a significant positive relationship with both COVID-19–induced strain (Model 1: *γ*=0.24, *P*<.001) and mHealth emergency use intention (Model 2: *γ*=0.28, *P*<.001). Results of model 3 showed that COVID-19–induced strain was significantly positively correlated with mHealth emergency use intention (*γ*=0.36, *P*<.001) when mHealth emergency use intention was simultaneously regressed on strain and event disruption. H1 and H2 were supported. The results are shown in Table 5.

To further explore the mediating role of COVID-19–induced strain on the temporal relationship between event disruption and mHealth emergency use intention, a Monte Carlo bootstrapping test was performed using R (version 3.5.3; R Foundation for Statistical Computing). Both the direct relationship (effect=0.18, 95% CI 0.06-0.30) and indirect relationship (effect=0.09, 95% CI 0.05-0.14) were significant. The results are summarized in Table 6. H3 was supported.

**Figure 2:**
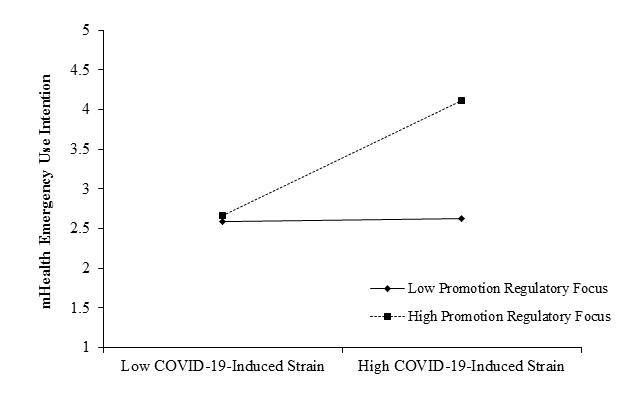
Moderating role of promotion regulatory focus. mHealth: mobile health.

The results for promotion regulatory focus are shown in model 4. The interaction of promotion regulatory focus with COVID-19–induced strain was positively associated with mHealth emergency use intention (*γ*=0.35, *P*=.02). To further explore the moderating role of promotion regulatory focus, we performed a Monte Carlo bootstrapping test. The moderating effect of promotion regulatory focus on the relationship between COVID-19–induced strain and mHealth emergency use intention was significantly stronger in the condition of high promotion regulatory focus (effect=0.49, 95% CI 0.34-0.65) than in the condition of low promotion regulatory focus (effect=0.25, 95% CI 0.09-0.50). The difference was also significant (effect=0.24, 95% CI 0.04-0.45), supporting H4. The moderating role of promotion regulatory focus is shown in Figure 2.

Finally, we used a Monte Carlo bootstrapping test to examine the moderated mediation model. The results showed that the indirect relationship between daily event disruption and mHealth emergency use intention through COVID-19–induced strain was significantly stronger when promotion regulatory focus was high (effect=0.12, 95% CI 0.06-0.19) than when it was low (effect=0.06, 95% CI 0.02-0.11). The difference between the two effects was significant (effect=0.06, 95% CI 0.001-0.12), supporting H5.

**Table 5:** Results of hierarchical linear model analysis.

Variables		COVID-19–induced strain			mHealth^a^ emergency use intention								
		Model 1^b^			Model 2^c^			Model 3^d^			Model 4^e^		
		*γ*	SE	*P* value	*γ*	SE	*P* value	*γ*	SE	*P* value	*γ*	SE	*P* value
Intercepts		3.43	0.21	<.001	4.23	0.30	<.001	4.22	0.31	<.001	4.20	0.31	<.001
**Between-person** **(** **N=110)**													
	Gender	–0.14	0.09	.115	–0.13	0.20	.51	–0.11	0.19	.55	–0.12	0.19	.55
	Education	0.09	0.04	<.001	0.08	0.09	.38	0.08	0.09	.38	0.08	0.09	.36
	Chronic disease	0.13	0.10	.20	0.31	0.14	.03	0.29	0.15	.06	0.29	0.15	.06
	Age	0.02	0.05	.59	0.04	0.08	.61	0.05	0.08	.52	0.05	0.08	.50
	Promotion regulatory focus	0.30	0.09	<.001	0.37	0.16	.02	0.43	0.16	.009	0.39	0.16	.02
**Within-person** **(** **N=550** **)**													
	Event disruption	0.24	0.05	<.001	0.28	0.06	<.001	0.19	0.06	<.001	0.18	0.06	.004
	COVID-19–induced strain	N/A^f^	N/A	N/A	N/A	N/A	N/A	0.36	0.06	<.001	0.37	0.06	<.001
**Interactive item**													
	COVID-19–induced strain × promotion regulatory focus	N/A	N/A	N/A	N/A	N/A	N/A	N/A	N/A	N/A	0.35	0.15	.02

^a^mHealth: mobile health.

^b^Pseudo *R*^2^=0.11.

^c^Pseudo *R*^2^=0.07.

^d^Pseudo *R*^2^=0.10.

^e^Pseudo *R*^2^=0.12.

^f^N/A: not applicable.

**Table 6:** Results of the Monte Carlo bootstrapping test.

Effect		Estimator	SE	95% CI^a^
**Moderating effect of promotion regulatory focus**				
	Low (M^b^ – SD)	0.25	0.08	0.09-0.40
	High (M + SD)	0.49	0.08	0.34-0.65
	Difference	0.24	0.11	0.04-0.45
**Mediating model of COVID-19–induced strain**				
	Direct effect	0.18	0.06	0.06-0.30
	Indirect effect	0.09	0.02	0.05-0.14
**Moderated mediation model**				
	Low (M – SD)	0.06	0.02	0.02-0.11
	High (M + SD)	0.12	0.03	0.06-0.19
	Difference	0.06	0.03	0.01-0.12

^a^Bootstrapping=20,000.

^b^M: mean.

## Discussion

We developed a conceptual model to explore how daily event disruptions of the COVID-19 pandemic predict mHealth emergency use intention. This study provides key findings and contributions to both mHealth research and practitioners.

### Principal Findings

This study presents three significant key findings. First, event disruptions of the COVID-19 pandemic are associated with increased daily mHealth emergency use intentions. Event disruptions of the COVID-19 pandemic represent the discontinuity of daily routines [2], resulting in the need for individuals to adapt their behaviors and attitudes [4]. During the COVID-19 pandemic, a major change has been an increased concern for disease prevention [54]. However, the uncertainty of the SARS-CoV-2 virus has driven people to find a means to access immediate medical information and health care services in urgent situations [55], for which mHealth may be a solution. Given the fluctuations of event disruptions caused by the COVID-19 pandemic, this study also explores the temporal influences of event disruption on mHealth emergency use intention. The results found that individuals increased their intention to use mHealth to help deal with the event disruptions of the COVID-19 pandemic.

Second, this study found that COVID-19–induced strain mediated the relationship between event disruption and mHealth emergency use intention on a daily basis. According to CATS, stressful experiences or strain arises from the lack of resources to effectively deal with the demands of stressful events [56]. The SARS-CoV-2 virus is novel and highly infectious, putting high levels of stress on the public in general, which also threatens their mental health [57]. Consequently, individuals have a need for finding ways to alleviate this strain. Our research found that COVID-19–induced strain strengthens the mechanism through which event disruption facilitates mHealth emergency use intention.

Third, our research findings suggest that promotion regulatory focus amplifies the indirect effect of event disruption on mHealth emergency use intention through daily COVID-19–induced strain. The interaction of personality traits and contextual variables determines the choice of coping behavior [31]. When confronted with a stressful experience, individuals who are striving to maintain their health are likely to seek tools to acquire relevant medical information [58]. This is consistent with our finding that promotion regulatory focus plays a contingent role in the association between COVID-19–induced strain and mHealth emergency use intention.

### Theoretical Implications

This study provides several theoretical contributions to mHealth literature. First, this study contributes to the mHealth literature by identifying the temporal influences of event disruption and mHealth emergency use intention. The COVID-19 pandemic was used as an example situation to explore the influence of event disruption caused by an emergent health crisis on the use of mHealth. This study extended this line of research by not only incorporating event disruption as an influencing factor for mHealth emergency use intention but also by examining mHealth emergency use intention on a daily basis. In doing so, this study contributes to mHealth literature by identifying a new type of mHealth use intention and examining its proximal antecedent.

Second, our study uncovered an underlying mechanism by examining the mediating role of COVID-19–induced strain. Previous studies investigating mHealth use intention mainly focused on the influences of technological and psychological factors [8,59]. For instance, Hoque [60] found a positive relationship between perceived usefulness and mHealth use intention, while Liu et al [8] found that perceived autonomy increased the use of mHealth. This study extends this line of research concerning the influences of psychological characteristics by incorporating COVID-19–induced strain. We identified COVID-19–induced strain as a contributor to mHealth emergency use intention. Furthermore, our exploration of the impact of event disruption on strain showed that changes in the external environment increased individuals’ health concerns, associating with elevated stress levels. Individuals were likely to use mHealth for health self-management and to reduce strain. We have extended prior research by identifying the role of strain in shaping mHealth emergency use intention and gaining a better understanding of how a public health crisis impacts personal strain and mHealth emergency use intention.

Third, we have also enriched the understanding of CATS by incorporating promotion regulatory focus into our model. Previous research using CATS primarily focused on the role of expectations in shaping an individual’s response to stressful events [11]. The expected outcome is what motivates an individual to adopt certain coping strategies [61]. However, little is known about the criteria for evaluating specific expected outcomes. We propose that promotion regulatory focus could be a possible explanation. For individuals with high promotion regulatory focus, the expected outcome would be to maintain personal health when confronted with psychological strain caused by the COVID-19 pandemic. We found that promotion regulatory focus prompts individuals to rely more on mHealth to cope with psychological strain in a health emergency. This provides useful insight into the formation process of expectations.

### Practical Implications

This study has practical implications for mHealth providers during a public health crisis. When the public experiences a health crisis, many people use mHealth services, which helps deal with psychological strain. We recommend that service providers develop specific services to cater to the needs of the public. For instance, remote primary diagnosis and health monitoring for a specific disease can be integrated into mHealth. This would enable individuals to incorporate mHealth into their daily lives and allow effective self-monitoring, even in urgent situations.

In addition, it would be useful for service providers to consider the role of regulatory focus, as individuals with high promotion regulatory focus are more likely to use mHealth when confronted with a health emergency. Service providers may adopt the regulatory focus scale as a primary screening method to select potential users and provide them with specific functions and services. This would offer providers with opportunities to increase user compliance.

### Limitations and Future Research

This study has several limitations and points out directions for future research. First, we did not establish a causal relationship between event disruption and mHealth emergency use intention. Moreover, although we collected two-wave data on a daily basis, we cannot conclude that daily event disruption predicts psychological strain and mHealth emergency use intention because we did not manipulate the event disruptions of the COVID-19 pandemic. Future research could use a cross-lagged panel design to infer the causal relationship between event disruption and mHealth use intention.

Second, it is also not possible to rule out common method variance. The experience sampling method controls for common method variance to a certain degree, as confirmed by the multilevel confirmatory factor analysis. However, our data were collected through self-report questionnaires, and therefore, our results may still have been impacted by common method variance. Future research could acquire objective data to minimize the potential effects of common method variance. This could be implemented through gathering mHealth app browsing history during a public health emergency.

Another limitation is that our study was conducted in China. Further research is needed to test the generalizability of our findings in other countries. The development of the mHealth industry differs across the world and mHealth use will depend on the stage of development of this technology. Therefore, future research in other countries will need to additionally consider these factors.

### Conclusions

mHealth provides individuals with a platform to access health care services. The results showed that event disruption of the COVID-19 pandemic induced mHealth emergency use intention through increased psychological strain. Furthermore, individuals’ promotion regulatory focus amplified this indirect relationship. Our findings extend our understanding of the factors underlying mHealth emergency use intention and illustrate the potential contingent role of promotion regulatory focus in CATS. This study also opens avenues for future research on mHealth emergency use intention in other countries and cultural settings.

## Appendix

Multimedia Appendix 1 Questionnaire: measurement of the major constructs.

## Abbreviations

CATS: cognitive activation theory of stress
H: hypothesis
HLM: hierarchical linear modeling
mHealth: mobile health
