# Supplementary material for: COVID-19–Related Disruptions and Increased mHealth Emergency Use Intention: Experience Sampling Method Study
Source: JMIR Mhealth Uhealth. 2020 Dec 30;8(12):e20642. doi: 10.2196/20642 (PMC7775377; doi:10.2196/20642)
Supplement: Multimedia Appendix 1 [file mhealth_v8i12e20642_app1.docx]

Daily Measurements

Event Disruption (Morgeson, Mitchell, & Liu, 2015)

ED1: Today, COVID-19 pandemic disrupted my ability to get its work done;

ED2: Today, COVID-19 pandemic caused me to stop and think about how to respond;

ED3: Today, COVID-19 pandemic altered my normal way of responding;

ED4: Today, COVID-19 pandemic required me to change the way it does its work.

COVID-19 Induced Strain (House & Rizzo, 1972)

CIS1: Due to COVID-19 Pandemic, I lived and worked under a great deal of tension today.

CIS2: I felt fidgety or nervous as a result of COVID-19 pandemic today.

CIS3: Problems associated with life and work caused by COVID-19 pandemic may keep me awake at night today.

mHealth Emergency Use Intention (Liu, Ngai, & Ju, 2019)

EUI1: Today, I intended to use mHealth services under urgent medical requirements

EUI2: Today, I predicted to use mHealth services under urgent medical requirements.

EUI3: Today, I planned to use mHealth services when I am in urgent need of medical care.

Baseline Measurement

Promotion Regulatory Focus (Lockwood, Jordan, & Kunda, 2002)

PRF1: I frequently imagine how I will achieve my hopes and aspirations.

PRF2: I often think about the person I would ideally like to be in the future

PRF3: I typically focus on the success I hope to achieve in the future

PRF4: I often think about how I will achieve academic success.

PRF5: My major goal in school right now is to achieve my academic ambitions

PRF6: I see myself as someone who is primarily striving to reach my “ideal self”—to fulfill my hopes, wishes, and aspirations

PRF7: In general, I am focused on achieving positive outcomes in my life.

PRF8: I often imagine myself experiencing good things that I hope will happen to me.

PRF9: Overall, I am more oriented toward achieving success than preventing failure.
